# Thermodynamics of the Vapor–Liquid–Solid Growth of Ternary III–V Nanowires in the Presence of Silicon

**DOI:** 10.3390/nano11010083

**Published:** 2021-01-02

**Authors:** Hadi Hijazi, Mohammed Zeghouane, Vladimir G. Dubrovskii

**Affiliations:** 1 Faculty of Laser Photonics and Optoelectronics, ITMO University, Kronverkskiy prospect 49, 197101 Saint Petersburg, Russia; 2 CNRS, SIGMA Clermont, Institut Pascal, Université Clermont Auvergne, F-63000 Clermont-Ferrand, France; mohammed.zeghouane@uca.fr; 3 Faculty of Physics, St. Petersburg State University, Universitetskaya Emb. 13B, 199034 St. Petersburg, Russia; dubrovskii@mail.ioffe.ru

**Keywords:** vapor–liquid–solid, nanowires, ternary alloys, silicon, doping

## Abstract

Based on a thermodynamic model, we quantify the impact of adding silicon atoms to a catalyst droplet on the nucleation and growth of ternary III–V nanowires grown via the self-catalyzed vapor–liquid–solid process. Three technologically relevant ternaries are studied: InGaAs, AlGaAs and InGaN. For As-based alloys, it is shown that adding silicon atoms to the droplet increases the nanowire nucleation probability, which can increase by several orders magnitude depending on the initial chemical composition of the catalyst. Conversely, silicon atoms are found to suppress the nucleation rate of InGaN nanowires of different compositions. These results can be useful for understanding and controlling the vapor–liquid–solid growth of ternary III–V nanowires on silicon substrates as well as their intentional doping with Si.

## 1. Introduction

Epitaxial growth of ternary III–V compounds [1] enables delicate bandgap engineering which is paramount for optoelectronic applications [2,3,4,5,6]. However, the compositional tuning of ternary thin films is often limited by misfit dislocations due to strain induced by the lattice mismatch between (i) elemental binaries forming a ternary alloy, and/or (ii) the grown film and the underlying substrate such as silicon [7,8,9]. Lattice mismatch between the two binaries is the main reason for their strong interactions in the solid phase, leading to the so-called miscibility gaps in InGaAs and InGaN [1].

Due to their high surface-to-volume ratio and small footprint with the substrate, nanowire (NW) structures allow for a very efficient strain relaxation at the free lateral facets [10,11,12,13]. This property opens a way for nearly unlimited compositional tuning in ternary III–V NWs, including some material combinations that can hardly be achieved in thin films, as well as dislocation-free growth of such NWs on silicon substrates [14,15,16,17].

Most III–V NWs are epitaxially grown by the vapor–liquid–solid (VLS) mechanism in which the solid phase forms from a supersaturated liquid alloy in a metal catalyst droplet [18]. The latter can be made of either a foreign metal, often gold [18], or a group III metal (Ga) in the self-catalyzed VLS process [15]. In Au-catalyzed VLS growth of ternary III–V NWs, the liquid alloy in the droplet is quaternary, while it becomes ternary in the self-catalyzed process. In addition, a small fraction of foreign species, such as Si atoms, can also be added to the droplet. This can be achieved intentionally when doping of NWs is required [19,20,21,22,23], or unintentionally when a catalyst droplet absorbs foreign atoms from the underlying substrate. The latter process often occurs in the initial nucleation step of III–V NWs grown on (oxidized) silicon substrates, and is enhanced at higher temperatures [24,25,26]. Intentional or unintentional Si doping is supposed to not only affect the electrical and optical properties of III–V NWs, but also to change their growth mechanism itself. Adding Si atoms into a catalyst droplet may influence the NW nucleation and growth rates, morphology, and structural properties. It is well-known that chemical potential driving the liquid–solid transition strongly depends on the liquid composition [27,28] and hence on the Si concentration in the droplet. We have already reported how the presence of Si atoms influences the nucleation of some binary III–V and III–N NWs [29,30], but this has not been done for ternaries so far.

Consequently, here we develop an analytical model to describe the influence of Si doping on the nucleation rate of InGaAs, AlGaAs, and InGaN NWs of different chemical compositions. We only consider Si impurities because (i) it is one of the most commonly used dopants for III–V materials, and (ii) the VLS growth of ternary III–V NWs on Si is important for monolithic integration of photonics with the existing electronic platform. However, our approach should apply for other doping elements and III–V ternaries.

## 2. Model

**Figure 1:**
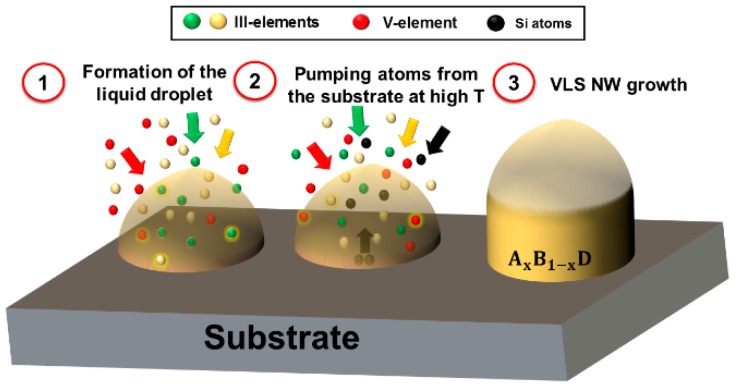
Schematic representation of the vapor–liquid–solid (VLS) system studied in this work. Stage 1: a group III liquid droplet absorbs two types of group III atoms and group V atoms from vapor. Stage 2: Si atoms are added to the droplet from the substrate (by either diffusion or through evaporation and re-condensation on the droplet surface at high T), or by the direct impingement from vapor. Stage 3: the VLS growth of a ternary $A_{x}B_{1-x}D$ NW from a droplet containing Si atoms.

We consider a ternary III–V NW growing via the VLS process from a liquid catalyst droplet which contains, in addition to the three growth species, a small fraction of Si atoms $c_{\mathrm{Si}}$, as shown in Figure 1. Our approach is based on the calculation of the change of chemical potential difference between the liquid and solid phases induced by the presence of Si atoms. For the nucleation and growth of a ternary $A_{x}B_{1-x}D$ from a supersaturated liquid phase containing, generally, A, B, D, Au, and Si atoms (and no Au in the case of self-catalyzed NWs), the difference of chemical potentials between liquid and solid can be written as

(1) $$\Delta \mu =x\left(\mu_{A}^{L}+\mu_{D}^{L}-\mu_{\mathrm{AD}}^{S}\right)+\left(1-x\right)\left(\mu_{B}^{L}+\mu_{D}^{L}-\mu_{\mathrm{BD}}^{S}\right).$$

The chemical potential $\mu_{q}^{L}$ of component q in liquid is given by

(2) $$\mu_{q}^{L}=\mu_{q}^{\mathrm{PL}}+\ln\left(c_{s}\right)+\frac{1}{2}\sum_{i,j=1}^{m}c_{i}c_{j}\left(\omega_{\mathrm{is}}+\omega_{\mathrm{js}}-\omega_{\mathrm{ij}}\right),$$

and the chemical potentials of AD and BD pairs in solid write

(3) $$\mu_{\mathrm{AD}}^{S}=\mu_{\mathrm{AD}}^{\mathrm{PS}}+\ln x+\omega_{S}{\left(1-x\right)}^{2},$$

(4) $$\mu_{\mathrm{BD}}^{S}=\mu_{\mathrm{BD}}^{\mathrm{PS}}+\ln(1-x)+\omega_{\mathrm{AD}-\mathrm{BD}}^{S}x^{2}.$$

Here, $\mu_{q}^{\mathrm{PL}}$, $\mu_{\mathrm{AD}}^{\mathrm{PS}}$, and $\mu_{\mathrm{BD}}^{\mathrm{PS}}$ are the chemical potentials of atoms in pure liquid and III–V pairs in pure solid binaries, respectively, which depend only on temperature T [31,32], m is the number of growth elements present in the droplet, $\omega_{\mathrm{AD}-\mathrm{BD}}^{s}$ is the temperature-dependent interaction parameter between AD and BD pairs in solid, and $\omega_{\alpha \beta }$ are the compositional-dependent pseudo-binary interaction parameters between α and β atoms in liquid [33]. We measure all chemical potentials and interaction parameters in thermal units of $k_{B}T$, with $k_{B}$ as the Boltzmann constant.

After adding Si atoms to the droplet, the initial atomic fraction $c_{q}^{0}$ of element q must be reduced by the factor ($1-c_{\mathrm{Si}}$), that is, $c_{q}=c_{q}^{0}\left(1-c_{\mathrm{Si}}\right)$ [29,30]. In the present study, we do not consider any possible influence of Si dopant on the chemical potential of solid NW. Therefore, the system can be described only by the change of chemical potentials of different atoms in liquid. Generalizing the result of Reference [30] to the case of ternaries, we find

(5) $$\begin{array}{c} \rho ={x\rho }_{\mathrm{AD}}+\left(1-x\right)\rho_{\mathrm{BD}}=x\underset{q=A,D\;}{\sum }\left(\mu_{q}^{L}-\mu_{q}^{0,L}\right)+\left(1-x\right)\underset{q=B,D\;}{\sum }\left(\mu_{q}^{L}-\mu_{q}^{0,L}\right)\\ \;\;\;\;\;\;\;\;\;\;=2\ln\left(1-c_{\mathrm{Si}}\right)+x\underset{q=A,D}{\sum }\left(\lambda_{q}c_{\mathrm{Si}}^{2}+\xi_{q}c_{\mathrm{Si}}\right)+\left(1-x\right)\underset{q=B,D}{\sum }\left(\lambda_{q}c_{\mathrm{Si}}^{2}+\xi_{q}c_{\mathrm{Si}}\right), \end{array}$$

with

(6) $$\lambda_{q}=\frac{1}{2}\overset{m}{\underset{i,j=1}{\sum }}c_{i}^{0}c_{j}^{0}\left(\omega_{i,x}+\omega_{j,q}-\omega_{i,j}\right)-\overset{m}{\underset{i=1}{\sum }}c_{i}^{0}\left(\omega_{i,q}+\omega_{\mathrm{Si},q}-\omega_{i,\mathrm{Si}}\right)+\omega_{\mathrm{Si},q}$$

(7) $$\xi_{q}=-\overset{m}{\underset{i,j=1}{\sum }}c_{i}^{0}c_{j}^{0}\left(\omega_{i,q}+\omega_{j,q}-\omega_{i,j}\right)+\overset{m}{\underset{i=1}{\sum }}c_{i}^{0}\left(\omega_{i,q}+\omega_{\mathrm{Si},q}-\omega_{i,\mathrm{Si}}\right)$$

ρ, $\rho_{\mathrm{AD}}$ and $\rho_{\mathrm{BD}}$ are the changes of the liquid phase supersaturation induced by adding Si atoms for ternary, AD and BD binaries, respectively.

Due to the exponential dependence of the nucleation probability P for each NW monolayer formation on Δμ, a small fluctuation of the liquid chemical potentials results in an extremely steep change of P. This change is given by [29]

(8) $$P\;≅\exp\left(i_{c}\rho \right)$$

where $i_{c}$ is the critical nucleus size of classical nucleation theory and hence must be much larger than unity. The probability P determines the nucleation rate of NWs emerging from catalyst droplet and the axial growth rate of developed NWs. According to Equation (8), our model allows one to describe the influence of Si on the nucleation and growth of ternary III–V NWs by calculating the function ρ given by Equations (5)–(7). This function contains the solid composition x, which is why the self-consistent determination of ρ requires a relationship between the compositions of ternary NW and the liquid droplet.

According to Reference [28], the composition of a ternary NW can be determined by minimizing the corresponding formation energy F with respect to x

(9) $$\frac{\partial F}{\partial x}=\frac{\partial \Delta \mu }{\partial x}i+\frac{\partial a}{\partial x}i^{1/2}=0$$

Here, $i=i_{\mathrm{AD}}+i_{\mathrm{BD}}$ is the total number of III–V pairs in a growing two-dimensional nucleus (fractional monolayer) and a is a surface energy constant. It is commonly assumed that the surface energy should be independent of the composition of the nucleus itself, because the solid compound with lower surface energy segregates at the nucleus boundary [28]. Therefore, the surface energy constant a can be considered independent of x. This leads to the simplified form of Equation (9) given by

(10) $$\frac{\partial \Delta \mu }{\partial x}=0,$$

meaning that the solid composition corresponds to the minimum of chemical potential.

Using Equation (1) in Equation (10), we obtain the relation between the liquid composition $y=c_{A}/\left(c_{A}+c_{B}\right)$ (which simply equals the ratio of the concentration of element a over the total group III concentration in the droplet) and x in the form

(11) φ(y)=Φ(x)+Γ(T),

with

(12) $$\varphi \left(y\right)=\ln\left(\frac{y}{1-y}\right)-2\omega_{\mathrm{AD}}\left(1-c_{D}\right)y,$$

(13) $$\Phi \left(x\right)=\ln\left(\frac{x}{1-x}\right)+\omega_{\mathrm{AD}-\mathrm{BD}}^{s}\left(1-x\right)$$

(14) $$\Gamma \left(T\right)=\;\Delta \mu_{\mathrm{AD}}^{0}-\Delta \mu_{\mathrm{BD}}^{0}+{\left(1-c_{D}\right)}^{2}\omega_{\mathrm{AB}}+c_{D}^{2}\left(\omega_{\mathrm{AD}}-\omega_{\mathrm{BD}}\right)+c_{D}\left(1-c_{D}\right)\left[\omega_{\mathrm{AB}}+\omega_{\mathrm{AD}}-\omega_{\mathrm{BD}}\right]$$

Unfortunately, no analytical solution to Equation (11) can be obtained unless we assume a negligible atomic fraction of the group V element D in the liquid phase, which is not relevant for all systems. For example, ${\mathrm{In}}_{x}{\mathrm{Ga}}_{1-x}\mathrm{As}$ NWs can be grown by hydride vapor phase epitaxy (HVPE) only at high temperatures T ≥ 950 K. According to Reference [31], the As concentration in such NWs reaches ~11% in an almost pure In droplet. Consequently, Equation (11) should generally be solved numerically in order to precisely determine the compositional diagram y(x) at high group V concentrations.

## 3. Results and Discussion

We now apply the model to the three important ternary III–V NWs. In calculations, we consider self-catalyzed VLS NWs, for which the droplet contains only the growth species and Si. The first-order interaction parameters between the solid binaries used in calculations are listed in Table 1. For the temperature-dependent InAs-GaAs interaction parameter, temperature should be taken in K.

### 3.1. InGaAs System

**Figure 2:**
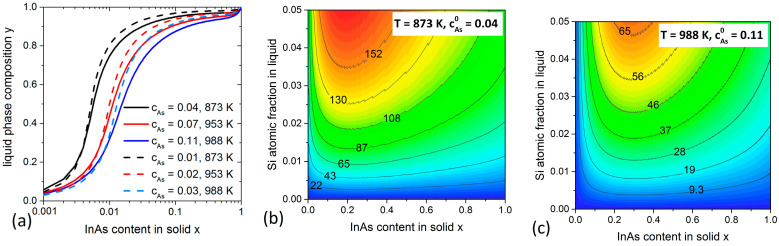
Calculations for ${\mathrm{In}}_{x}{\mathrm{Ga}}_{1-x}\mathrm{As}$ system. (**a**) Compositional diagrams y(x) at different T. The initial As concentrations $c_{\mathrm{As}}^{0}$ are estimated from the In-As binary phase diagram [31]. (**b**,**c**) Contour plots of $\rho \left(c_{\mathrm{Si}},x,T\right)$ (in meV) at T= 872 K and 988 K.

We start our calculations with ${\mathrm{In}}_{x}{\mathrm{Ga}}_{1-x}\mathrm{As}$ NWs growth at different temperatures T. Figure 2a shows the evolution of the liquid composition y required to form the NW of composition x at a fixed As atomic fraction $c_{\mathrm{As}}$ and different T. The real $c_{\mathrm{As}}$ values are expected to vary quite considerably depending on whether the droplet is In- or Ga-rich according to the respective phase diagrams [31]. Therefore, two sets of values for $c_{\mathrm{As}}$ were chosen which correspond to pure In or pure Ga droplets. Despite the large difference in $c_{\mathrm{As}}$, the calculated y(x) curves reveal that the catalyst droplet consists mainly of In (y > 0.9) for the entire range of practical compositions of ${\mathrm{In}}_{x}{\mathrm{Ga}}_{1-x}\mathrm{As}$ alloy (0.1 < x ≤ 1). This correlates with what has been reported earlier for ${\mathrm{In}}_{x}{\mathrm{Ga}}_{1-x}\mathrm{As}$ NWs [28,36,37] and suggests that high In content in the liquid phase is thermodynamically required to grow solid ${\mathrm{In}}_{x}{\mathrm{Ga}}_{1-x}\mathrm{As}$. Consequently, in what follows we only use the $c_{\mathrm{As}}$ values corresponding to pure In droplet. The temperatures considered are above the critical temperature of 816 K [28], which is why no miscibility gap is present in Figure 2a. The relationship between the solid and liquid compositions can now be used to quantify the influence of adding Si atoms to the catalyst droplet on the nucleation probability of InGaAs NW monolayers. We fix only temperature T (and hence $c_{\mathrm{As}}^{0}$), then the ρ function can be calculated using the y values deduced for each x. The atomic fraction of Si is varied up to 0.05. The results are shown in the contour plots in Figure 2b,c for two different T. The numbers given on the solid level lines represent different values of $\rho \left(c_{\mathrm{Si}},x,T\right)$, expressed in meV. The calculated ρ values are always positive, showing that the presence of Si in In-rich catalyst droplets is beneficial for nucleation of ${\mathrm{In}}_{x}{\mathrm{Ga}}_{1-x}\mathrm{As}$ NWs regardless of their composition x, including almost pure GaAs solid (x→0). This is different from the results reported in Reference [29], where pure GaAs NWs were grown from pure Ga droplets. The effect is due to the presence of In in the catalyst droplet which, even at small x, may have a great impact on the growth thermodynamics due to the interactions with the co-existing species in liquid. The highest values of ρ, corresponding to the largest enhancement of the nucleation and growth rates of InGaAs NWs, are obtained for compositions from 0.1 to 0.5 for both temperatures. The maximum of ρ decreases with increasing the growth temperature. It is about 160 meV at T = 872 K and decreases to 65 meV at T = 988 K. This high temperature is typical for growing InGaAs NWs by HVPE technique.

### 3.2. AlGaAs System

**Figure 3:**
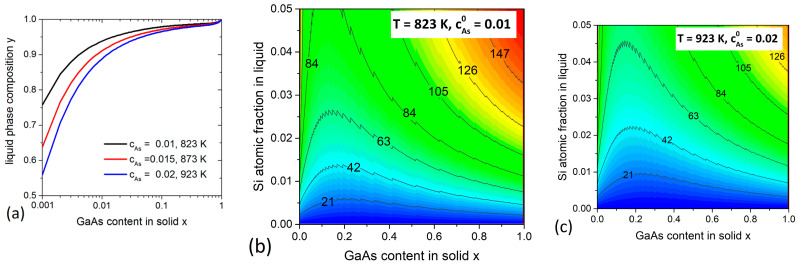
Calculations for ${\mathrm{Al}}_{1-x}{\mathrm{Ga}}_{x}\mathrm{As}$ system. (**a**) Compositional diagrams y(x) at different T. The initial As concentrations $c_{\mathrm{As}}^{0}$ are estimated from the Ga-As phase diagram [31]. (**b**,**c**) Contour plots of $\rho \left(c_{\mathrm{Si}},x,T\right)$ (in meV) at T= 823 K and 923 K.

We now consider the self-catalyzed VLS growth of ${\mathrm{Al}}_{1-x}{\mathrm{Ga}}_{x}\mathrm{As}$ NWs. The liquid phase of this VLS system is known to consist mainly of Ga [27]. Therefore, the As atomic fraction can be estimated from the Ga-As binary phase diagram [31]. For the typical growth temperatures, this concentration varies between 0.01 and 0.02 [33]. Note that, similar to GaAs, growth regimes with even lower $c_{\mathrm{As}}$ may also be possible [38]. Figure 3a shows the variation of y with x for different growth temperatures. The values of y are close to unity in the compositional range of x between 0.05 and 1, and remain quite large (>0.6) for even lower x. This confirms that very low Al contents in the liquid phase are sufficient to tune the AlAs fraction in the solid alloy from 0.05 to 1, while almost pure AlAs NWs can grow from a liquid droplet with y = 0.6–0.8 [27,28]. The impact of Si on the nucleation rate of AlGaAs NW monolayers is shown in Figure 3b,c for two different T. The trend is the same as for InGaAs system—the ρ values increase with increasing the Si fraction in the droplet. However, the highest values of ρ are now located in the Ga-rich zones, where the maximum reaches about 150 at T= 823 K (MBE conditions) and 125 meV at 923 K (HVPE conditions).

### 3.3. InGaN System

Finally, we investigate ${\mathrm{In}}_{x}{\mathrm{Ga}}_{1-x}N$ NWs which can be grown by HVPE via the self-catalyzed VLS mode [39]. This material can cover the whole range of visible light spectrum by tuning its chemical composition toward larger x. However, the large lattice mismatch between InN and GaN (~11%) leads to segregation of In inside the miscibility gap [12] and planar growth of homogeneous In-rich InGaN alloys remains challenging. Therefore, a significant effort is being put to fabricate InGaN material in the form of NWs to reduce the miscibility gap. It has been experimentally demonstrated that the miscibility gap can be circumvented in the HVPE growth regimes with high degrees of supersaturation of a mother phase, either in In-Ga droplets or in the vapor phase in the catalyst-free selective area epitaxy [39,40]. It is therefore important to investigate thermodynamics of the VLS InGaN NWs within our approach. We consider the growth conditions at different T corresponding to different growth techniques. Unfortunately, the solubility of N in liquid In-Ga alloy is unknown. Therefore, we fix $c_{N}^{0}$ fixed at the plausible values of 0.05 and 0.1 for the calculations of y(x), but only at 0.05 for the calculation of ρ.

**Figure 4:**
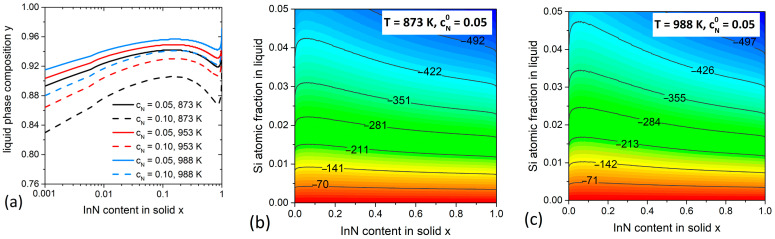
Calculations for ${\mathrm{In}}_{x}{\mathrm{Ga}}_{1-x}N$ system. (**a**) Compositional diagrams y(x)  at different T and $c_{N}^{0}$, where the wavy regions correspond to the miscibility gaps. (**b**,**c**) Contour plots of $\rho \left(c_{\mathrm{Si}},x,T\right)$ (in meV) at T= 873 K and 988 K.

The miscibility gap corresponds to the wavy regions in the y(x) curves in Figure 4a within the range of x between ~0.2 and ~0.8. It is slightly reduced at higher T. One can see that a high In concentration in liquid (y > 0.88) is always required to obtain any relevant x. The impact of adding Si to such In-rich droplets is shown in Figure 4b,c. Unlike As-based ternaries, the supersaturation is significantly reduced in the presence of Si. A small amount of Si of only about 0.005 is enough to reduce the chemical potential of InGaN in the liquid phase by 70 meV, which induces a significant decrease of the nucleation probability P. Further increase of $c_{\mathrm{Si}}$ can raise the ρ values to ~500 meV. Furthermore, the growth temperature is found to have almost no impact on the curves in Figure 4b,c. We can thus conclude that adding Si to the catalyst droplets has negative impact on the nucleation and growth of VLS InGaN NWs with whatever composition, and hence the initial supersaturation must be very high to enable the formation of such NWs on Si substrates.

## 4. Conclusions

In conclusion, a thermodynamic study is presented to quantify the change of chemical potential in the liquid phase and the corresponding nucleation rate of ternary NW monolayers in the self-catalyzed VLS process influenced by the presence of Si atoms in a catalyst droplet. We have investigated three technologically relevant systems: ${\mathrm{In}}_{x}{\mathrm{Ga}}_{1-x}\mathrm{As}$, ${\mathrm{Al}}_{x}{\mathrm{Ga}}_{1-x}\mathrm{As}$ and ${\mathrm{In}}_{x}{\mathrm{Ga}}_{1-x}N$. Before calculating the evolution of chemical potential with $c_{Si}$, we determined the relationship between the liquid and solid phase concentrations of the growth species at a given temperature. The results appear very different for the As- and N-based NWs. For the As-based ternaries, the presence of Si increases the nucleation rate of ternary NWs, while nucleation of ${\mathrm{In}}_{x}{\mathrm{Ga}}_{1-x}N$ NWs is significantly reduced. Our model is not restricted to these systems and can be extended to study other ternary III–V NWs growing by the self-catalyzed or Au-catalyzed VLS process.

## Figures and Tables

**Table 1:** Interaction parameters of solid binaries in InGaAs, AlGaAs, and InGaN.

Ternary System	Interaction Parameter $\omega^{s}$ (eV)	Reference
InAs-GaAs	0.2042 + 7.8 × 10^−5^ T	[34]
AlAs-GaAs	0.027	[27]
InN-GaN	0.3035	[35]
